# Insights into the bacterial community and its temporal succession during the fermentation of wine grapes

**DOI:** 10.3389/fmicb.2015.00809

**Published:** 2015-08-18

**Authors:** Hailan Piao, Erik Hawley, Scott Kopf, Richard DeScenzo, Steven Sealock, Thomas Henick-Kling, Matthias Hess

**Affiliations:** ^1^Department of Viticulture and Enology, Washington State UniversityRichland, WA, USA; ^2^ZeaChem Inc.Boardman, OR, USA; ^3^Pacific Rim WinemakersWest Richland, WA, USA; ^4^ETS LaboratoriesSaint Helena, CA, USA; ^5^Functional Systems Microbiology Laboratory, University of California, DavisDavis, CA, USA; ^6^Department of Energy Joint Genome InstituteWalnut Creek, CA, USA

**Keywords:** wine bacteria, wine fermentation, temporal succession, organic grape products, 16S rRNA gene profile, next-generation sequencing

## Abstract

Grapes harbor complex microbial communities. It is well known that yeasts, typically *Saccharomyces cerevisiae*, and bacteria, commonly the lactic acid fermenting *Oenococcus oeni*, work sequentially during primary and secondary wine fermentation. In addition to these main players, several microbes, often with undesirable effects on wine quality, have been found in grapes and during wine fermentation. However, still little is known about the dynamics of the microbial community during the fermentation process. In previous studies culture dependent methods were applied to detect and identify microbial organisms associated with grapes and grape products, which resulted in a picture that neglected the non-culturable fraction of the microbes. To obtain a more complete picture of how microbial communities change during grape fermentation and how different fermentation techniques might affect the microbial community composition, we employed next-generation sequencing (NGS)—a culture-independent method. A better understanding of the microbial dynamics and their effect on the final product is of great importance to help winemakers produce wine styles of consistent and high quality. In this study, we focused on the bacterial community dynamics during wine vinification by amplifying and sequencing the hypervariable V1–V3 region of the 16S rRNA gene—a phylogenetic marker gene that is ubiquitous within prokaryotes. Bacterial communities and their temporal succession was observed for communities associated with organically and conventionally produced wines. In addition, we analyzed the chemical characteristics of the grape musts during the organic and conventional fermentation process. These analyses revealed distinct bacterial population with specific temporal changes as well as different chemical profiles for the organically and conventionally produced wines. In summary these results suggest a possible correlation between the temporal succession of the bacterial population and the chemical wine profiles.

## Introduction

Wine is an alcoholic beverage that is produced by fermenting grapes and represents a heterogeneous mixture of complex compounds. Many of the wines' compounds contribute to their characteristic color, aroma, and flavor (Styger et al., 2011; González-Barreiro et al., 2015), and are released during the fermentation process. The metabolic conversion of grape juice into wine is a complex process of alcoholic fermentation and malolactic fermentation (MLF) and involves a mixture of different microorganisms (Fugelsang and Edwards, 2007). Yeasts play important roles during the alcoholic fermentation step and have significant impact on wine quality. Although bacteria are not the main driving force behind wine characteristics and quality, they do have a significant effect on the final product. For example, lactic acid bacteria are known to convert L-malic acid to lactic acid through MLF and to impart flavor complexity, while acetic acid bacteria (AAB) produce acetic acid, which is a key factor in wine spoilage. MLF is important in winemaking by regulating deacidification and microbial stability. MLF usually occurs after the alcoholic fermentation but it may occur during the alcoholic fermentation process. It is possible that monitoring bacterial community profiles during alcoholic fermentation might allow predicting and controlling wine quality more efficiently. Microorganisms that are present during the various stages of vinification have significant impact on the wine quality both positively and negatively (Fleet, 1993; Fugelsang and Edwards, 2007). To ensure consistent high quality wines and allow reliable risk management, it is essential to monitor the microbial populations throughout the vinification process. NGS represents a fast and precise approach to obtain high-resolution insights into the population dynamics.

In past years, several microorganisms have been found in association with wine grapes and wine musts using culture-dependent techniques (Cappello et al., 2004). These conventional microbiology methods facilitated the isolation of a number of yeasts (e.g., *Brettanomyces/Dekkera, Issatchenkia, Zygoascus*, and *Zygosaccharomyces*) (Curtin et al., 2007; Barata et al., 2012; Di Toro et al., 2015), AAB (e.g., *Acetobacter and Gluconacetobacter*) (Barata et al., 2012), and lactic acid bacteria (e.g., *Enterococcus, Lactobacillus, Lactococcus, Oenococcus*, and *Pediococcus*) (Beneduce et al., 2004; Bae et al., 2006; Capozzi et al., 2010; Garofalo et al., 2015). Due to the viable but non-culturable nature of many wine microorganisms or the dominance of a few organisms that grow very well under laboratory conditions, these conventional microbiology approaches resulted in a rather incomplete and biased picture of the microbial community that is involved in the fermentation process (Millet and Lonvaud-Funel, 2000; Oliver, 2005; Cocolin et al., 2013). In more recent years, a culture-independent method called PCR-DGGE, which combines polymerase chain reaction (PCR) with denaturing gradient gel electrophoresis (DGGE), has been frequently used for detecting specific microorganisms during different stages of the wine fermentation process (Renouf et al., 2007; Spano et al., 2007; Andorrá et al., 2008; Laforgue et al., 2009; Pérez-Martín et al., 2014). Although PCR-DGGE remains a useful tool to detect and discriminate microbial organisms potentially present in wine grapes and musts without cultivation, it has its limitation due to the challenge of distinguishing co-migrating bands from multiplexed PCR products and requirement of intensive bands (Laforgue et al., 2009; Cocolin et al., 2013). With next-generation sequencing (NGS) technologies being a commodity now, powerful tools for high-throughput analysis of complex microbial communities via amplification and subsequent sequencing of the 16S ribosomal RNA (rRNA) hypervariable regions are now available (Sinclair et al., 2015). NGS have been applied widely and resulted in new insights into microbial community dynamics from diverse environmental samples (Piao et al., 2014; Trexler et al., 2014; Nguyen and Landfald, 2015; Pessoa-Filho et al., 2015) including grape and botrytized wine (Bokulich et al., 2012, 2014), but it is still not well known how the microbial communities associated with different grapes change over time and how these changes affect the final quality of the fermentation products.

There has been a fast growing demand for organic foods and beverages and the market for organically produced wines has experienced a significant boost. To obtain an enhanced understanding of how the different winemaking techniques affect bacterial community dynamics and further find out the bacterial community dynamics affect wine fermentation, we analyzed the temporal succession of the bacterial community and its effects on the changes of chemical characteristics during organic and conventional wine fermentation using 16S rRNA amplicon sequencing. The obtained results revealed a broad bacterial diversity in wine including known wine bacteria. Many of the identified organisms have to our knowledge not been reported to date. By analyzing the dynamics of the bacterial population during the fermentation process, it was possible to detect bacteria that were previously not associated with wine fermentation. The chemical characteristics of the wines, combined with the results of bacterial community profiles, indicated that there might be a possible link between specific bacteria, their succession and some wine characteristics.

## Materials and methods

### Sample collection

Both organic and conventional pied-de-cuve (PDC) were obtained by stomping and fermenting hand-harvested organically grown Riesling grapes in a 200 gallon tote. No sulfur dioxide (SO_2_) was added to the organic PDC fermentation, whereas SO_2_ (55.8 mg/L) was added during the conventional PDC fermentation process. For organic and conventional bulk fermentation, the organically grown Riesling grapes were machine pressed and transferred to a 15,000 gallon fermentation tank. Juice was allowed to settle for 36 h before heavy solids were removed. When sugar content of the organic or conventional PDC reached approximately 10 Brix, the PDCs were transferred to bulk fermentation tanks. Fermentation temperature was maintained between 10 and 13°C. Neither SO_2_ nor fining agents were added to the organic musts during primary fermentation, while SO_2_ (38.5 mg/L) and bentonite were added to the conventional musts. Yeast assimilable nitrogen was added in the form of autolyzed yeast product and diammonium phosphate (DAP) to the organic and conventional wine respectively. Brix and ethanol measurements were taken to monitor fermentation progress and fermentation was terminated when a Brix of 2.5 and 6.9 was reached for organic and conventional wine, respectively.

### DNA extraction and 16S rRNA gene amplification

Total microbial DNA was extracted from 500 mg of the organic and conventional wine samples using a FastDNA SPIN Kit for Soil (MP Biomedical, Solon, OH) according to the manufacturer's instructions. Extracted DNA was quantified with a spectrophotometer (Nanodrop ND1000; Thermo Scientific, USA). The hypervariable V1–V3 region of the 16S rRNA gene was amplified from the environmental DNA using the primer set 28F/519R (28F: 5′-ccatct catccctgcgtgtctccgactcagxxxxxxxxGAGTTTGATCNTGG CTCAG-3′ and 519R: 5′-cctatcc cctgtgtgccttggcagtctcagGTNTTACNGCGGCKGC TG-3′). Primer sequences were modified by the addition of 454 A or B adapter sequences (lower case) and ended with the sequencing key “TCAG” (underlined). In addition, the forward primer included a 8 bp barcode, indicated by xxxxxxxx in the forward primer sequence above, for multiplexing of samples during sequencing. The barcode sequence for each sample is listed in Table S1.

The V1–V3 region of the 16S rRNA genes was amplified with primer pair 28F/519R by emulsion PCR. Subsequent PCR reactions were performed using the Roche Live amplification mix (according to the Roche protocol) with the following PCR conditions: initial denaturation for 1 min at 94°C, followed by 50 amplification cycles of (30 s at 94°C, 4.5 min at 58°C, and 30 s at 68°C), and hold at 10°C. Emulsion PCR and sequencing of the PCR amplicons were performed following the Roche 454 GS FLX Titanium technology instructions provided by the manufacturer.

### Data analysis

Raw pyrosequencing data were demultiplexed and processed using QIIME version 1.7.0 (Caporaso et al., 2010b). Sequencing primers and barcodes were removed from the raw sequence reads by allowing 1.5 mismatches to the barcode and 2 mismatches to the primer sequence. Sequences were removed if they had homopolymeric regions of more than 6 nt, were smaller than 200 nt, had quality scores lower than 25, or if they were identified as being chimeric. This resulted in a total of 16,142 and 28,490 high quality 16S rRNA gene sequences from organic and conventional wine samples, respectively.

Quality filtered sequences were clustered into operational taxonomic units (OTUs) at a 97% sequence identity cut-off using UCLUST (Edgar, 2010). The most abundant sequence of each OTU was picked as representative sequence. Singleton and doubleton abundance, Shannon, Simpson, and Chao1 estimators were calculated using the QIIME software. Representative sequences were aligned using the PyNAST algorithm (Caporaso et al., 2010a) and the alignment was filtered to remove common gaps. Following the quality filtering and grouping steps, 1340 unique sequences (representing 44,632 total sequences) were aligned and taxonomically classified using the RDP classifier program (Wang et al., 2007) with 80% confidence rating against the Greengenes database (McDonald et al., 2012).

### Chemical analysis

Chemical analyses of the wine samples were performed at ETS Laboratories (Saint Helena, CA) using an Agilent 7700 inductively coupled plasma-mass spectrometer according to manufacturer's instructions and as described by Hopfer et al. (2013).

## Results

### Bacterial community profile of organically and conventionally produced wine

**Figure 1:**
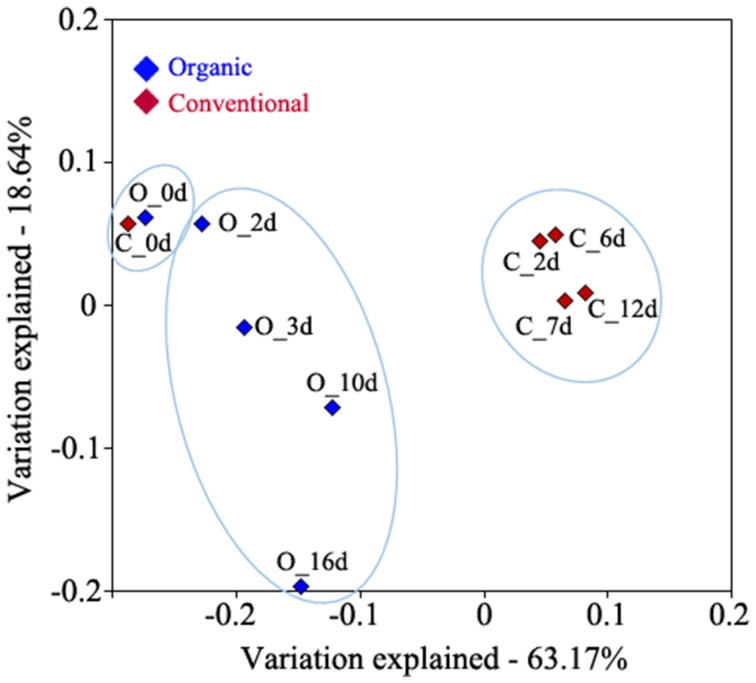
**Principal component analysis of 16S rRNA data from microbiomes associated with grape must during the fermentation process**. 16S rRNA amplicon data was generated from PDC (O_0d and C_0d) and during organic (O_2d, O_3d, O_10d, and O_16d) and conventional (C_2d, C_6d, C_7d, and C_12d) bulk fermentation. The percentage of variation explained by the plotted principal coordinates is indicated on the axes.

To determine bacterial community dynamics and their effects on wine components, we compared the profiles of the bacterial community in wines that were produced using organic and conventional fermentation protocols. Grape juice was inoculated with indigenous yeasts from the grape skins by adding PDC. This traditional wine making technique reduces the needs for commercial yeast and usually increases wine complexity. Samples for bacterial community profiling were collected from the PDC (0 day) and must at different fermentation stages after PDC was added to the grape juice. Environmental DNA was extracted from PDC and must followed by pyrosequencing of the hypervariable V1–V3 region of the 16S rRNA genes. The quality-filtered pyrotag reads were clustered into OTUs at a 97% of sequence identity level, which resulted in 529 and 1099 distinct OTUs, representing 16,142 and 28,490 sequences from organic and conventional wine, respectively (Table 1). Analysis of OTUs profiles suggests that community richness within organic wine was stable at early stage of fermentation (0, 2, and 3 days; Table 1; Table S2). Continuing the fermentation process, increased community richness at 10 days was measured, whereas decreased community richness was observed afterwards (Table 1; Table S2). Compared to organically producing wine, bacterial community richness increased significantly at 6 days of fermentation (Table 1; Table S2) then decreased rapidly within 24 h (Table 1; Table S2) during conventional wine production. These findings are supported by the calculated rarefaction curves (Figure S1). Shannon's diversity and Simpson indices are higher in conventionally fermented wine (Table S2), suggesting that the bacterial community in conventionally produced wine became more diverse than in organically produced wine. Principal component analysis suggests that the wine microbiome profiles associated with grape must during conventional fermentation were distinct from the microbiome profiles associated with grape must from organic fermentation (Figure 1).

**Table 1:** **Summary of generated reads and OTUs observed**.

**Duration of fermentation [days]**	**Organic**						**Conventional**					
	**0**	**2**	**3**	**10**	**16**	**Total**	**0**	**2**	**6**	**7**	**12**	**Total**
Quality filtered reads	5,420	3,569	4,188	1,583	1,382	16,142	16,001	1,531	7,588	2,127	1,243	28,490
OTUs observed	173	165	176	202	146	529	268	201	612	220	160	1099

### Phylogenetic profiles of the bacterial communities during the fermentation processes

**Figure 2:**
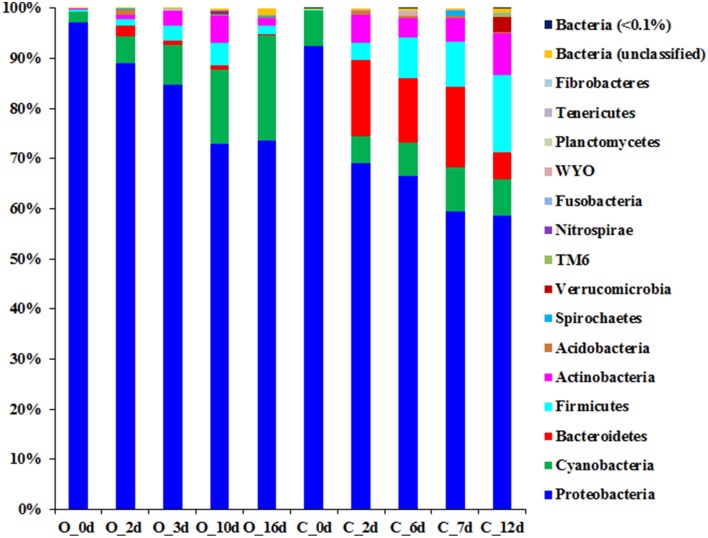
**Phylogenetic profile of microbiomes associated with grape must during the fermentation process**. Phylogeny was assigned in the phylum level based on the RDP database after quality-filtered reads were clustered using 97% sequence identity cut-off.

Clustering of the obtained 16S rRNA gene sequences based on a 97% sequence identity cut-off and assigning phylogeny to each of the obtained OTUs suggest that a total of 15 phyla (contributing ≥1 of the reads) were present during the fermentation process of the two grape musts under observation (Figure 2 and Table S3). Nine of the observed 15 phyla were found in musts from both fermentation techniques (i.e., *Proteobacteria, Cyanobacteria, Bacteroidetes, Firmicutes, Actinobacteria, Acidobacteria, Spirochaetes, Verrucomicrobia*, and *Fusobacteria*), while the presence of some phyla depended on the applied fermentation technique. Specifically, *Nitrospirae, Planctomycetes*, and *Tenericutes* were detected solely in the samples from organically fermented must, whereas *Fibrobacteres* and members of the candidate phylum WYO were detected only in the conventionally produced wine musts (Figure 2 and Table S3). It is possible that members of these specific phyla might contribute to the distinct chemical characteristics of the produced wines. *Proteobacteria* is the predominant phylum in both wine musts (Figure 2 and Table S3), which was represented primarily by the *Gammaproteobacteria* within the PDC (0 day). During fermentation the relative abundance of *Gammaproteobacteria* decreased significantly in both wine musts (6–8 fold), which was partially complemented by an increase of other members of the *Proteobacteria*, i.e., *Alphaproteobacteria, Betaproteobacteria*, and *Deltaproteobacteria* (Table 2). During organic fermentation, the abundance of *Alphaproteobacteria* increased and this phylogenetic group became the dominant class (57% at 15 days). During conventional fermentation, population of *Alphaproteobacteria* increased as well (~4.5 fold) but did not dominate the community (21.72–27.63%). Abundance of *Betaproteobacteria* increased 250–380 fold to a relative abundance between 18.15 and 27.10% (Table 2). Overall population changes suggest a notable reduction of *Proteobacteria* (Figure 2 and Table S3), which is similar to what has been observed previously during botrytized wine fermentation (Bokulich et al., 2012). This decrease in *Proteobacteria*, specifically of the *Gammaproteobacteria*, was accompanied by an increase of the *Bacteroidetes, Firmicutes*, and *Actinobateria*. The increase was in particular notable within the microbiome from the conventionally fermented wine, while the increase was less notable within the microbiome from organically fermented wine (Table 2 and Table S3). Within the conventionally fermented wine, the increase of abundance of *Bacteroidetes* was caused through a significant increase in *Spingobacteriia* and a moderate increase in *Bacteroidia* (Figure 2; Table 2 and Table S3). The increase of *Firmicutes* was due largely to an increase of the *Bacilli* and a moderate increase of the *Clostridia* (Table 2). Further analysis of the bacterial community resulted in the detection of 96 genera across all samples, of which 33 genera were found both in organically and conventionally fermented must. Twenty-one of the 96 genera were detected only within the bacterial communities associated with organically fermented must, whereas 42 genera were found only within the bacterial communities associated with conventionally fermented grapes (Table 3). Increased genus diversity was observed for the microbiome from conventionally fermented must (75 genera total) when compared to the microbiome from organically fermented must (54 genera total). Representatives of the genus *Gluconobacter*, an acetic acid bacterium commonly found associated with grape skin (Joyeux et al., 1984), was detected in the microbiome of both wine types, however discrete changes within the *Gluconobacter* population were observed between organically and conventionally fermented wines. Comparison between organically and conventionally produced wines revealed that the population of *Gluconobacter* was highly abundant in organic PDC fermentation (8.67% at 0 day), while it possessed very low abundance in conventional PDC fermentation (0.47% at 0 day; Table 3). During the fermentation process, the *Gluconobacter* population increased in both musts and eventually represented the predominant genus from organically produced wine at late stage (49%; 16 day), while it was relatively stable, accounting for 5–7% of population, throughout the conventional fermentation process (5–7%; Table 3). Beside the dominant genus *Gluconobacter*, a number of other genera (total sequences detected >1% in data from at least one of the time points) were also detected during both fermentation procedures (i.e., *Clavibacter, Propionibacterium, Hymenobacter, Pedobacter, Bacillus, Staphylococcus, Acetobacter, Spingomonas, Diaphorobacter, Janthinobacterium, Ralstonia, Neisseria, Acinetobacter, Pseudomonas*, and *Leptospira*), with *Pedobacter, Spingomonas, Janthinobacterium*, and *Pseudomonas* exhibiting dominance only during the conventional fermentation process (Table 3). In addition, other less abundant phylogenetic groups (total sequences detected between 0.1 and 1%) were observed during the two distinct fermentation processes (i.e., *Corynebacterium, Micrococcus, Sediminibacterium, Dyadobacter, Exiguobacterium, Lactobacillus, Clostridium, Roseburia, Faecalibacterium, Fusobacterium, Bradyrhizobium, Methylobacterium, Roseomonas, Salinispora, Curvibacter, Pelomonas, Trabulsiella*, and *Haemophilus*) (Table 3). Interestingly, *Oenococcus*, a genus containing known lactic acid bacteria, was detected only in the microbiome of conventionally fermented wine (Table 3).

**Table 2:** **Relative abundance of prokaryotes associated with grape musts during organic and conventional fermentation at the class level**.

**Duration of fermentation [days]**	**Organic**					**Conventional**				
	**0**	**2**	**3**	**10**	**16**	**0**	**2**	**6**	**7**	**12**
Acidobacteria;c_Acidobacteria-2	0.00	0.90	0.00	0.38	0.29	0.00	0.72	0.46	0.33	0.00
Actinobacteria;c_Actinobacteria	0.06	0.98	2.96	5.31	1.52	0.05	5.68	3.58	4.84	8.21
Actinobacteria;c_Thermoleophilia	0.00	0.00	0.00	0.00	0.00	0.00	0.00	0.13	0.00	0.00
Bacteroidetes;c_Bacteroidia	0.04	0.03	0.05	0.32	0.00	0.01	2.55	1.74	2.77	0.24
Bacteroidetes;c_Flavobacteriia	0.00	0.73	0.00	0.13	0.00	0.00	0.26	0.58	0.47	0.00
Bacteroidetes;c_Sphingobacteriia	0.02	1.37	0.72	0.38	0.14	0.02	12.48	10.46	12.60	5.15
Cyanobacteria;c_4C0d-2	0.04	0.03	0.00	0.00	0.00	0.00	0.13	0.91	0.00	0.00
Cyanobacteria;c_S15B-MN24	0.02	0.11	0.31	0.63	0.43	0.00	0.98	0.61	1.13	2.65
Cyanobacteria;c_Synechococcophycideae	0.00	0.00	0.00	1.01	0.00	0.00	0.00	0.00	0.00	0.00
Fibrobacteres;c_Fibrobacteria	0.00	0.00	0.00	0.00	0.00	0.00	0.00	0.18	0.00	0.00
Firmicutes;c_Bacilli	0.44	0.98	2.65	3.79	1.52	0.19	1.44	6.80	5.88	10.62
Firmicutes;c_Clostridia	0.02	0.17	0.43	0.76	0.22	0.06	1.96	1.32	3.10	4.83
Fusobacteria;c_Fusobacteria	0.00	0.00	0.00	0.13	0.00	0.01	0.00	0.00	0.00	0.16
Nitrospirae;c_Nitrospira	0.07	0.00	0.00	0.32	0.00	0.00	0.00	0.00	0.00	0.00
Planctomycetes;c_Planctomycetia	0.00	0.00	0.19	0.00	0.00	0.00	0.00	0.00	0.00	0.00
Proteobacteria;c_Alphaproteobacteria	12.49	9.78	24.52	30.32	57.16	5.82	27.63	22.26	23.93	21.72
Proteobacteria;c_Betaproteobacteria	0.06	4.82	3.15	7.14	2.53	0.07	22.73	27.10	18.15	23.65
Proteobacteria;c_Deltaproteobacteria	0.11	0.31	0.00	0.44	0.43	0.02	0.00	0.29	1.50	1.93
Proteobacteria;c_Gammaproteobacteria	84.59	74.05	57.07	34.87	13.46	86.56	18.68	16.75	15.84	11.34
Spirochaetes;c_Leptospirae	0.00	0.22	0.05	0.19	0.14	0.00	0.07	0.00	1.18	0.00
Tenericute;c_Mollicutes	0.00	0.00	0.14	0.00	0.00	0.00	0.00	0.03	0.00	0.00
TM6;c_SJA-4	0.00	0.00	0.05	0.06	0.07	0.00	0.00	0.00	0.00	0.72
Verrucomicrobia;c_Opitutae	0.00	0.00	0.00	0.00	0.00	0.00	0.00	0.00	0.00	3.06
Verrucomicrobia;c_Verruco-5	0.00	0.00	0.00	0.13	0.00	0.00	0.00	0.00	0.00	0.00

**Table 3:** **Relative abundance of prokaryotes associated with grape musts during organic and conventional fermentation at the genus level**.

**Duration of fermentation [days]**	**Organic**					**Conventional**				
	**0**	**2**	**3**	**10**	**16**	**0**	**2**	**6**	**7**	**12**
Actinobacteria;c_Actinobacteria;o_Actinomycetales;f_Corynebacteriaceae;g_Corynebacterium	0.00	0.00	0.79	0.69	0.29	0.01	0.39	0.18	0.24	0.48
Actinobacteria;c_Actinobacteria;o_Actinomycetales;f_Dermabacteraceae;g_Brachybacterium	0.00	0.00	0.00	0.00	0.00	0.00	0.20	0.00	0.00	0.00
Actinobacteria;c_Actinobacteria;o_Actinomycetales;f_Dietziaceae;g_Dietzia	0.00	0.00	0.00	0.00	0.14	0.01	0.00	0.00	0.00	0.00
Actinobacteria;c_Actinobacteria;o_Actinomycetales;f_Gordoniaceae;g_Gordonia	0.00	0.00	0.00	1.45	0.00	0.00	0.00	0.00	0.00	0.24
Actinobacteria;c_Actinobacteria;o_Actinomycetales;f_Microbacteriaceae;g_Clavibacter	0.00	0.00	0.19	0.32	0.00	0.00	0.20	0.00	0.00	1.53
Actinobacteria;c_Actinobacteria;o_Actinomycetales;f_Microbacteriaceae;g_Curtobacterium	0.00	0.08	0.00	0.00	0.00	0.00	0.20	0.67	0.47	0.00
Actinobacteria;c_Actinobacteria;o_Actinomycetales;f_Microbacteriaceae;g_Frigoribacterium	0.00	0.00	0.00	0.00	0.00	0.00	0.26	0.00	0.00	0.00
Actinobacteria;c_Actinobacteria;o_Actinomycetales;f_Microbacteriaceae;g_Leucobacter	0.00	0.00	0.00	0.00	0.00	0.00	0.00	0.00	1.03	0.00
Actinobacteria;c_Actinobacteria;o_Actinomycetales;f_Microbacteriaceae;g_Microbacterium	0.00	0.34	0.05	0.00	0.00	0.00	0.00	0.05	0.00	0.00
Actinobacteria;c_Actinobacteria;o_Actinomycetales;f_Microbacteriaceae;g_Rathayibacter	0.00	0.00	0.00	0.00	0.00	0.00	0.26	0.18	0.00	0.00
Actinobacteria;c_Actinobacteria;o_Actinomycetales;f_Microbacteriaceae;g_Salinibacterium	0.00	0.00	0.00	0.00	0.00	0.00	0.46	0.12	0.00	0.00
Actinobacteria;c_Actinobacteria;o_Actinomycetales;f_Micrococcaceae;g_Kocuria	0.00	0.00	0.00	0.00	0.14	0.00	0.00	0.00	0.00	0.00
Actinobacteria;c_Actinobacteria;o_Actinomycetales;f_Micrococcaceae;g_Micrococcus	0.00	0.00	0.00	0.76	0.00	0.00	0.33	0.11	0.28	0.08
Actinobacteria;c_Actinobacteria;o_Actinomycetales;f_Mycobacteriaceae;g_Mycobacterium	0.00	0.08	0.00	0.06	0.00	0.00	0.00	0.03	0.00	0.56
Actinobacteria;c_Actinobacteria;o_Actinomycetales;f_Nocardiaceae;g_Rhodococcus	0.00	0.00	0.26	0.00	0.00	0.00	0.07	0.00	0.00	0.00
Actinobacteria;c_Actinobacteria;o_Actinomycetales;f_Propionibacteriaceae;g_Propionibacterium	0.06	0.48	0.55	1.52	0.43	0.03	0.65	1.75	1.97	4.51
Bacteroidetes;c_Bacteroidia;o_Bacteroidales;f_Bacteroidaceae;g_Bacteroides	0.04	0.00	0.00	0.00	0.00	0.01	0.07	0.03	0.47	0.00
Bacteroidetes;c_Bacteroidia;o_Bacteroidales;f_Prevotellaceae;g_Prevotella	0.00	0.03	0.00	0.00	0.00	0.00	0.52	0.37	0.71	0.16
Bacteroidetes;c_Bacteroidia;o_Bacteroidales;f_[Paraprevotellaceae];g_[Prevotella]	0.00	0.00	0.00	0.00	0.00	0.00	0.20	0.01	0.05	0.00
Bacteroidetes;c_Flavobacteriia;o_Flavobacteriales;f_Flavobacteriaceae;g_Chryseobacterium	0.00	0.00	0.00	0.06	0.00	0.00	0.26	0.46	0.47	0.00
Bacteroidetes;c_Sphingobacteriia;o_Sphingobacteriales;f_Chitinophagaceae;g_Sediminibacterium	0.00	0.00	0.00	0.13	0.00	0.00	0.00	0.21	0.09	0.80
Bacteroidetes;c_Sphingobacteriia;o_Sphingobacteriales;f_Chitinophagaceae;g_Segetibacter	0.00	0.00	0.00	0.00	0.00	0.00	0.00	0.04	0.47	0.00
Bacteroidetes;c_Sphingobacteriia;o_Sphingobacteriales;f_Flexibacteraceae;g_Dyadobacter	0.00	0.28	0.00	0.19	0.00	0.00	0.00	0.17	0.00	0.00
Bacteroidetes;c_Sphingobacteriia;o_Sphingobacteriales;f_Flexibacteraceae;g_Hymenobacter	0.00	0.56	0.10	0.00	0.00	0.00	1.05	1.79	1.74	1.53
Bacteroidetes;c_Sphingobacteriia;o_Sphingobacteriales;f_Flexibacteraceae;g_Spirosoma	0.00	0.00	0.00	0.00	0.00	0.00	0.00	0.18	0.00	0.00
Bacteroidetes;c_Sphingobacteriia;o_Sphingobacteriales;f_Sphingobacteriaceae;g_Pedobacter	0.00	0.50	0.62	0.00	0.14	0.00	9.67	7.39	8.56	2.33
Cyanobacteria;c_Synechococcophycideae;o_Synechococcales;f_Synechococcaceae;g_Prochlorococcus	0.00	0.00	0.00	1.01	0.00	0.00	0.00	0.00	0.00	0.00
Fibrobacteres;c_Fibrobacteria;o_Fibrobacterales;f_Fibrobacteraceae;g_Fibrobacter	0.00	0.00	0.00	0.00	0.00	0.00	0.00	0.18	0.00	0.00
Firmicutes;c_Bacilli;o_Bacillales;f_Bacillaceae;g_Anoxybacillus	0.00	0.00	0.00	0.06	0.00	0.00	0.00	0.03	0.33	0.00
Firmicutes;c_Bacilli;o_Bacillales;f_Bacillaceae;g_Bacillus	0.02	0.87	1.27	0.95	0.43	0.08	0.33	0.12	0.00	1.77
Firmicutes;c_Bacilli;o_Bacillales;f_Bacillaceae;g_Geobacillus	0.00	0.00	0.00	0.25	0.00	0.00	0.00	0.00	0.00	0.00
Firmicutes;c_Bacilli;o_Bacillales;f_Bacillaceae;g_Terribacillus	0.00	0.00	0.00	0.00	0.51	0.00	0.00	0.00	0.00	0.00
Firmicutes;c_Bacilli;o_Bacillales;f_Paenibacillaceae;g_Paenibacillus	0.00	0.00	0.00	0.06	0.14	0.00	0.00	0.09	0.00	0.00
Firmicutes;c_Bacilli;o_Bacillales;f_Staphylococcaceae;g_Staphylococcus	0.07	0.00	0.91	0.69	0.00	0.04	0.33	1.67	0.52	2.98
Firmicutes;c_Bacilli;o_Bacillales;f_Thermoactinomycetaceae;g_Planifilum	0.00	0.11	0.00	0.00	0.00	0.00	0.00	0.00	0.00	0.00
Firmicutes;c_Bacilli;o_Exiguobacterales;f_Exiguobacteraceae;g_Exiguobacterium	0.00	0.00	0.00	0.00	0.22	0.00	0.00	0.00	0.00	0.64
Firmicutes;c_Bacilli;o_Lactobacillales;f_Aerococcaceae;g_Aerococcus	0.00	0.00	0.00	0.19	0.00	0.00	0.00	0.00	0.00	0.00
Firmicutes;c_Bacilli;o_Lactobacillales;f_Lactobacillaceae;g_Lactobacillus	0.07	0.00	0.24	0.00	0.00	0.00	0.13	0.00	0.00	0.00
Firmicutes;c_Bacilli;o_Lactobacillales;f_Leuconostocaceae;g_Leuconostoc	0.04	0.00	0.00	0.00	0.00	0.00	0.00	0.00	0.00	0.00
Firmicutes;c_Bacilli;o_Lactobacillales;f_Leuconostocaceae;g_Oenococcus	0.00	0.00	0.00	0.00	0.00	0.00	0.00	1.83	4.00	0.88
Firmicutes;c_Bacilli;o_Lactobacillales;f_Leuconostocaceae;g_Weissella	0.00	0.00	0.00	0.13	0.00	0.00	0.00	0.00	0.00	0.00
Firmicutes;c_Bacilli;o_Lactobacillales;f_Streptococcaceae;g_Streptococcus	0.00	0.00	0.00	0.00	0.00	0.01	0.00	0.05	0.05	0.16
Firmicutes;c_Clostridia;o_Clostridiales;f_Clostridiaceae;g_Anaerococcus	0.00	0.00	0.00	0.00	0.00	0.00	0.00	0.00	0.00	3.30
Firmicutes;c_Clostridia;o_Clostridiales;f_Clostridiaceae;g_Clostridium	0.00	0.00	0.00	0.13	0.00	0.00	0.00	0.28	0.00	0.00
Firmicutes;c_Clostridia;o_Clostridiales;f_Lachnospiraceae;g_Blautia	0.00	0.00	0.00	0.00	0.00	0.01	0.00	0.01	0.28	0.00
Firmicutes;c_Clostridia;o_Clostridiales;f_Lachnospiraceae;g_Butyrivibrio	0.00	0.00	0.00	0.00	0.00	0.00	0.26	0.00	0.00	0.00
Firmicutes;c_Clostridia;o_Clostridiales;f_Lachnospiraceae;g_Moryella	0.00	0.00	0.00	0.00	0.00	0.00	0.00	0.00	0.00	0.16
Firmicutes;c_Clostridia;o_Clostridiales;f_Lachnospiraceae;g_Oribacterium	0.00	0.00	0.00	0.00	0.00	0.00	0.00	0.22	0.00	0.00
Firmicutes;c_Clostridia;o_Clostridiales;f_Lachnospiraceae;g_Roseburia	0.00	0.00	0.00	0.19	0.00	0.00	0.00	0.01	0.00	1.37
Firmicutes;c_Clostridia;o_Clostridiales;f_Peptococcaceae;g_Desulfosporosinus	0.00	0.00	0.00	0.00	0.14	0.00	0.00	0.00	0.00	0.00
Firmicutes;c_Clostridia;o_Clostridiales;f_Ruminococcaceae;g_Faecalibacterium	0.02	0.11	0.00	0.00	0.07	0.00	0.13	0.11	0.61	0.00
Firmicutes;c_Clostridia;o_Clostridiales;f_Ruminococcaceae;g_Oscillospira	0.00	0.00	0.02	0.00	0.00	0.00	0.00	0.16	0.33	0.00
Firmicutes;c_Clostridia;o_Clostridiales;f_Veillonellaceae;g_Megamonas	0.00	0.00	0.00	0.00	0.00	0.00	0.00	0.03	0.14	0.00
Firmicutes;c_Clostridia;o_Clostridiales;f_Veillonellaceae;g_Veillonella	0.00	0.00	0.00	0.00	0.00	0.00	0.33	0.00	0.09	0.00
Fusobacteria;c_Fusobacteria;o_Fusobacteriales;f_Fusobacteriaceae;g_Fusobacterium	0.00	0.00	0.00	0.13	0.00	0.00	0.00	0.00	0.00	0.16
Nitrospirae;c_Nitrospira;o_Nitrospirales;f_Nitrospiraceae;g_Nitrospira	0.07	0.00	0.00	0.32	0.00	0.00	0.00	0.00	0.00	0.00
Proteobacteria;c_Alphaproteobacteria;o_Rhizobiales;f_Bradyrhizobiaceae;g_Balneimonas	0.00	0.00	0.00	0.00	0.14	0.00	0.00	0.00	0.00	0.00
Proteobacteria;c_Alphaproteobacteria;o_Rhizobiales;f_Bradyrhizobiaceae;g_Bosea	0.00	0.00	0.00	0.00	0.00	0.00	0.00	0.36	0.00	0.00
Proteobacteria;c_Alphaproteobacteria;o_Rhizobiales;f_Bradyrhizobiaceae;g_Bradyrhizobium	0.00	0.00	0.00	0.88	0.14	0.00	0.00	0.16	0.00	0.00
Proteobacteria;c_Alphaproteobacteria;o_Rhizobiales;f_Methylobacteriaceae;g_Methylobacterium	0.00	0.28	0.00	0.76	0.07	0.00	0.33	0.82	0.00	0.40
Proteobacteria;c_Alphaproteobacteria;o_Rhizobiales;f_Phyllobacteriaceae;g_Phyllobacterium	0.00	0.00	0.29	0.00	0.00	0.00	0.00	0.00	0.00	0.00
Proteobacteria;c_Alphaproteobacteria;o_Rhizobiales;f_Rhizobiaceae;g_Agrobacterium	0.00	0.00	0.00	0.00	0.00	0.00	0.13	0.00	0.00	0.00
Proteobacteria;c_Alphaproteobacteria;o_Rhodospirillales;f_Acetobacteraceae;g_Acetobacter	0.00	0.00	0.29	0.00	0.29	0.00	0.65	0.32	2.07	0.24
Proteobacteria;c_Alphaproteobacteria;o_Rhodospirillales;f_Acetobacteraceae;g_Acidocella	0.00	0.00	0.00	0.00	0.00	0.00	0.26	0.00	0.00	0.00
Proteobacteria;c_Alphaproteobacteria;o_Rhodospirillales;f_Acetobacteraceae;g_Gluconobacter	8.67	3.28	19.56	17.50	49.42	0.47	7.32	6.10	7.57	5.63
Proteobacteria;c_Alphaproteobacteria;o_Rhodospirillales;f_Acetobacteraceae;g_Roseomonas	0.00	0.31	0.19	0.19	0.00	0.00	0.00	0.18	0.47	0.32
Proteobacteria;c_Alphaproteobacteria;o_Rhodospirillales;f_Rhodospirillaceae;g_Telmatospirillum	0.00	0.00	0.00	0.00	0.00	0.00	0.00	0.00	0.00	0.32
Proteobacteria;c_Alphaproteobacteria;o_Rickettsiales;f_Rickettsiaceae;g_Rickettsia	0.00	0.00	0.00	0.00	0.00	0.00	0.00	0.00	1.13	0.00
Proteobacteria;c_Alphaproteobacteria;o_Rickettsiales;f_Rickettsiaceae;g_Wolbachia	0.02	0.00	0.00	0.00	0.22	0.00	0.07	0.00	0.00	0.00
Proteobacteria;c_Alphaproteobacteria;o_Sphingomonadales;f_Sphingomonadaceae;g_Sphingomonas	0.02	1.01	0.41	0.95	0.80	0.00	8.43	9.83	6.58	6.60
Proteobacteria;c_Betaproteobacteria;o_Burkholderiales;f_Alcaligenaceae;g_Pigmentiphaga	0.00	0.00	0.00	0.00	0.00	0.00	0.07	0.08	0.42	0.00
Proteobacteria;c_Betaproteobacteria;o_Burkholderiales;f_Burkholderiaceae;g_Burkholderia	0.00	0.00	0.00	0.00	0.00	0.00	0.00	0.00	0.00	0.64
Proteobacteria;c_Betaproteobacteria;o_Burkholderiales;f_Burkholderiaceae;g_Salinispora	0.00	0.00	0.00	0.13	0.07	0.00	0.13	0.07	0.00	0.48
Proteobacteria;c_Betaproteobacteria;o_Burkholderiales;f_Comamonadaceae;g_Curvibacter	0.00	0.00	0.17	0.06	0.36	0.00	0.00	0.24	0.00	0.08
Proteobacteria;c_Betaproteobacteria;o_Burkholderiales;f_Comamonadaceae;g_Diaphorobacter	0.00	0.17	0.00	0.13	0.00	0.00	0.00	0.00	0.00	1.05
Proteobacteria;c_Betaproteobacteria;o_Burkholderiales;f_Comamonadaceae;g_Methylibium	0.00	0.00	0.00	0.00	0.00	0.00	0.07	0.30	0.56	0.00
Proteobacteria;c_Betaproteobacteria;o_Burkholderiales;f_Comamonadaceae;g_Pelomonas	0.00	0.00	0.14	0.00	0.00	0.00	0.59	0.00	0.00	0.00
Proteobacteria;c_Betaproteobacteria;o_Burkholderiales;f_Comamonadaceae;g_Ramlibacter	0.00	0.00	0.00	0.00	0.00	0.00	0.00	0.04	0.47	0.00
Proteobacteria;c_Betaproteobacteria;o_Burkholderiales;f_Comamonadaceae;g_Rubrivivax	0.00	0.00	0.00	0.00	0.00	0.00	0.13	0.25	0.00	0.00
Proteobacteria;c_Betaproteobacteria;o_Burkholderiales;f_Comamonadaceae;g_Schlegelella	0.00	0.00	0.00	0.00	0.00	0.00	0.00	0.13	0.00	0.00
Proteobacteria;c_Betaproteobacteria;o_Burkholderiales;f_Comamonadaceae;g_Variovorax	0.00	0.03	0.00	0.00	0.00	0.00	0.20	0.16	0.00	0.00
Proteobacteria;c_Betaproteobacteria;o_Burkholderiales;f_Oxalobacteraceae;g_Janthinobacterium	0.00	0.84	0.38	0.63	0.14	0.00	7.12	8.91	5.59	1.37
Proteobacteria;c_Betaproteobacteria;o_Burkholderiales;f_Oxalobacteraceae;g_Ralstonia	0.00	0.00	0.05	1.83	0.36	0.03	0.59	0.18	0.09	2.98
Proteobacteria;c_Betaproteobacteria;o_Neisseriales;f_Neisseriaceae;g_Neisseria	0.00	0.00	0.10	0.25	0.00	0.00	0.13	0.00	0.00	1.05
Proteobacteria;c_Betaproteobacteria;o_Rhodocyclales;f_Rhodocyclaceae;g_KD1-23	0.00	0.00	0.00	0.00	0.00	0.00	0.26	0.00	0.00	0.00
Proteobacteria;c_Gammaproteobacteria;o_Alteromonadales;f_Shewanellaceae;g_Shewanella	0.00	0.00	0.00	0.13	0.00	0.00	0.00	0.00	0.00	0.00
Proteobacteria;c_Gammaproteobacteria;o_Enterobacteriales;f_Enterobacteriaceae;g_Candidatus Hamiltonella	0.00	0.00	0.00	0.00	0.00	0.00	0.26	0.00	0.00	0.00
Proteobacteria;c_Gammaproteobacteria;o_Enterobacteriales;f_Enterobacteriaceae;g_Citrobacter	0.00	0.28	0.00	0.00	0.00	0.00	0.00	0.04	0.05	0.00
Proteobacteria;c_Gammaproteobacteria;o_Enterobacteriales;f_Enterobacteriaceae;g_Erwinia	0.02	0.00	0.00	0.06	0.07	0.01	0.52	0.30	0.33	0.00
Proteobacteria;c_Gammaproteobacteria;o_Enterobacteriales;f_Enterobacteriaceae;g_Escherichia	0.00	0.00	0.00	0.00	0.00	0.00	0.00	0.01	0.24	0.00
Proteobacteria;c_Gammaproteobacteria;o_Enterobacteriales;f_Enterobacteriaceae;g_Trabulsiella	0.20	0.22	0.17	0.25	0.14	0.36	0.00	0.00	0.00	0.00
Proteobacteria;c_Gammaproteobacteria;o_Pasteurellales;f_Pasteurellaceae;g_Haemophilus	0.00	0.00	0.29	0.32	0.07	0.00	0.07	0.00	0.00	0.40
Proteobacteria;c_Gammaproteobacteria;o_Pseudomonadales;f_Moraxellaceae;g_Acinetobacter	0.00	0.20	0.14	0.00	0.14	0.00	0.33	1.16	3.06	4.99
Proteobacteria;c_Gammaproteobacteria;o_Pseudomonadales;f_Moraxellaceae;g_Enhydrobacter	0.00	0.00	0.00	0.00	0.00	0.00	0.20	0.12	0.00	0.00
Proteobacteria;c_Gammaproteobacteria;o_Pseudomonadales;f_Pseudomonadaceae;g_Pseudomonas	0.02	0.45	1.03	0.63	0.14	0.12	10.39	8.95	6.54	2.57
Spirochaetes;c_[Leptospirae];o_[Leptospirales];f_Leptospiraceae;g_Leptospira	0.00	0.22	0.05	0.19	0.14	0.00	0.07	0.00	1.18	0.00

### Chemical component analysis from organic and conventional wine

**Figure 3:**
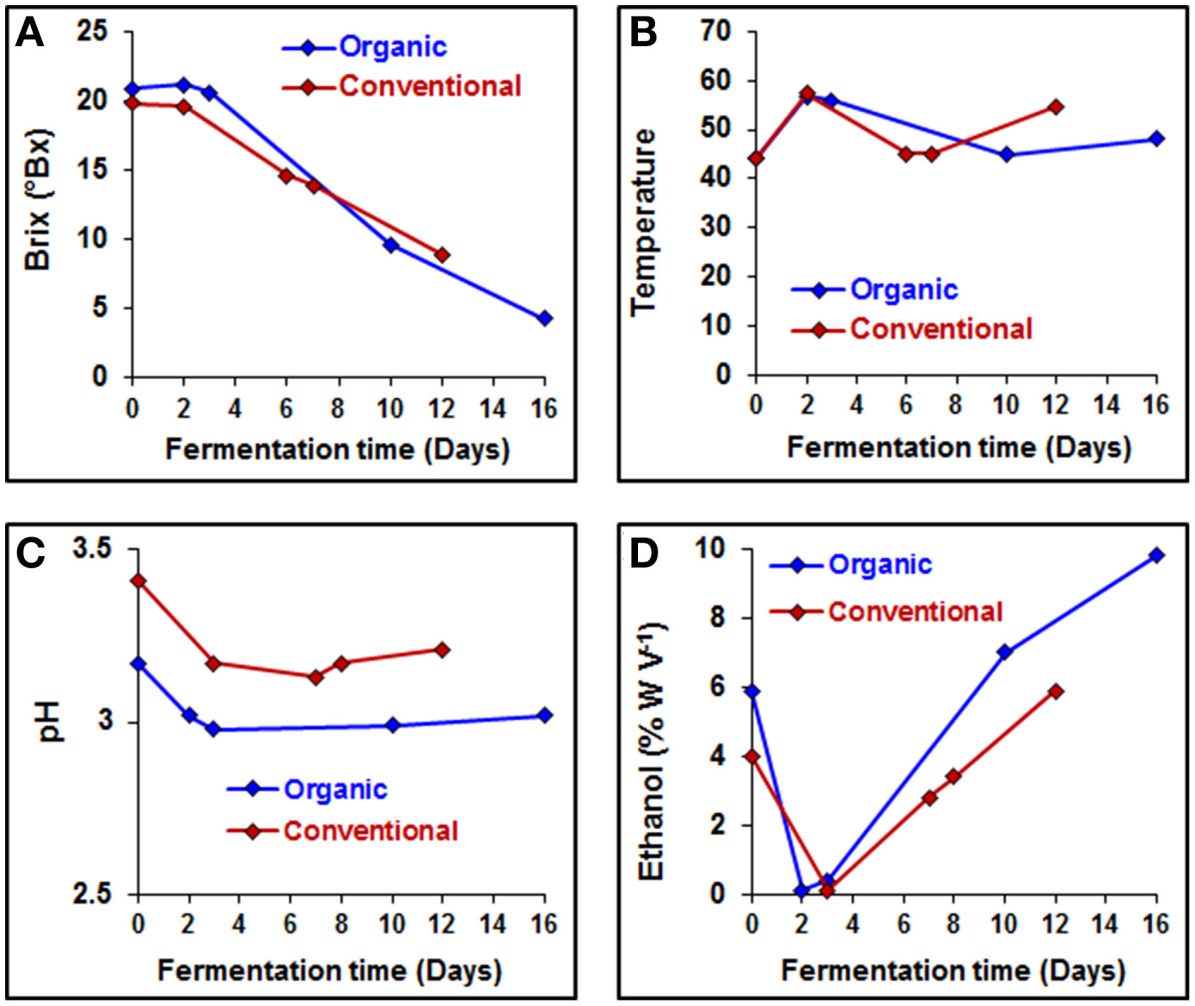
**Physicochemical characteristics of the organically and conventionally fermented grape musts**. **(A)** Fermentation rate (Brix), **(B)** fermentation temperature, **(C)** grape musts pH, and **(D)** production of ethanol were measured on each day of fermentation.

Several parameters, such as sugar concentration, temperature, pH value, ethanol concentration and a variety of chemical characteristics, of the grape must were monitored during the fermentation process (Figure 3 and Table 4). Sugar concentrations were stable until 3 days into the fermentation process, after this period sugar concentration decreased linearly in both wine fermentations (Figure 3A). Overall pH values were slightly lower from organically produced wine than conventionally produced wine, while ethanol reached a higher concentration during the organic fermentation process (Figures 3C,D). Lactic acid concentration at the end of the organic PDC fermentation was higher, while it was same in both wine fermentation processes, suggesting that wine fermentation was terminated before secondary fermentation was initiated. Malic acid content increased during both fermentation processes, however overall malic acid content was higher in conventionally fermented wine. Volatile acidity (VA) content changed irregularly, at early stage of fermentation (2–3 days) lower VA contents were measured for both types of wine samples, afterwards it increased to about three-fold in conventionally fermented wine, while it returned to first day level in organically fermented wine. Overall tartaric acid concentration was higher in organically fermented wine compare to conventionally fermented wine. A summary of the chemical characteristics of the grape musts is provided in Table 4. Initial nitrogen concentration was similar in both juices at the first day of fermentation and additional nitrogen was provided during the fermentation process to support continuous growth of yeast. Nitrogen concentrations are summarized in Table 4. More detailed and controlled studies will help to enhance our understanding of the molecular processes and microbe-microbe and microbe-must interaction would be of great value.

**Table 4:** **Chemical profile of grape musts during organic and conventional fermentation**.

**Duration of fermentation [days]**	**Organic**					**Conventional**				
	**0**	**2**	**3**	**10**	**16**	**0**	**2**	**6**	**7**	**12**
Ethanol at 20°C (% Vol)	5.9	0.1	0.4	7	9.8	4	0.1	2.8	3.4	5.9
L-lactic acid (g/L)	0.97	0.05	0.05	0.05	0.05	0.29	0.05	0.05	0.05	0.05
L-malic acid (g/L)	1.97	3.08	3.04	2.45	2.31	2.7	4.56	4.02	4.09	3.79
Volatile acidity(acetic) (g/L)	0.16	0.05	0.09	0.12	0.2	0.14	0.07	0.34	0.42	0.46
Tartaric acid (g/L)	2.1	4.2	4	3.2	3	1.7	2.5	2.1	1.9	1.9
Titratable acidity (g/L)	7.3	7.6	7.3	7.3	7.5	5.8	7.6	7.3	7.5	7.2
Yeast assimilable nitrogen (mg/L)	18	137	103	18	18	18	219	101	155	143
Alpha-amino compounds (as N) (mg/L)	10	91	70	14	12	14	112	54	56	59
Ammonia (mg/L)	10	56	40	10	10	10	130	57	120	102

## Discussion

Culture-independent NGS is a cost-effective approach to study composition and the spatial and temporal changes of microbial communities and it has been applied to various environment samples (Piao et al., 2014; Nguyen and Landfald, 2015). However, to our knowledge, as of today only a few studies have been published that employed NGS to study the dynamics of the microbial wine ecosystem (Bokulich et al., 2012, 2014, 2015). To enhance our understanding of the microbial dynamics, specifically of bacterial dynamics, during grape fermentation, we employed culture-independent 16S rRNA amplicon sequencing to determine changes in the bacterial population of grape must during the fermentation process. Currently, the most commonly used culture-independent method within the wine industry for comparing microbial populations associated with different grape products is PCR-DGGE (Cocolin et al., 2000; Lopez et al., 2003). PCR-DGGE possesses only a limited ability to provide detailed information about biodiversity within a sample as bands associated with different phylogenetic groups might be visible as a single band resulting in underestimation of microbial community diversity.

In this study we identified 96 genera and discriminated over 30 species that were present during wine fermentation. Importantly, most of the species we detected have not been reported previously during wine fermentation (Table S4), with the exception of a few species (i.e., *Propionibacterium acnes, Bacillus thermoamylovorans, Pseudomonas stutzeri*) that were isolated from grapevine, palm wine, and wine corks (Combet-Blanc et al., 1995; Bañeras et al., 2013; Yousaf et al., 2014). The genus *Gluconobacter* increased significantly during organic fermentation (from 3.28 to 49.42%), while it exhibited less notable changes during the conventional fermentation (from 5.63 to 7.57%) process (Table 3). A major difference of the organic and conventional wine making processes employed in this study was the addition of SO_2_ to the conventional wine prior to PDC fermentation (50 mg/L) and bulk fermentation (38.5 mg/L), while no SO_2_ added to the organic wine until completion of primary fermentation. The availability of SO_2_ during primary fermentation might represent a selective effect on the *Gluconobacter* population. Bokulich and colleagues showed that *Gluconobacter* population was significantly suppressed by SO_2_ at concentrations ≥25 mg/L (Bokulich et al., 2015). At higher taxonomic resolution the genus *Gluconobacter* was dominated by one distinct OTU (i.e., OTU denovo952) during the fermentation process (Table S5). To further define this specific OTU, its representing nucleotide sequence was compared to sequences deposited in NCBI database. Results revealed a 99.6% sequence identity with *Gluconobacter oxydans*, the main representative of AAB on grapes (Joyeux et al., 1984). *Gluconobacter oxydans* is known as spoilage acetic acid bacterium together with *Acetobacter* during winemaking; *Gluconobacter oxydans* is often detected in grapes, while *Acetobacter* is found in wine (Bartowsky and Henschke, 2008). Although AAB have been identified as wine spoilage bacteria previously, the population of AAB are often underestimated with culture-dependent method due to the lack of appropriate cultivation techniques (Millet and Lonvaud-Funel, 2000). Amplicon sequencing data allowed us to observe significant population changes of *Gluconobacter oxydans* during wine fermentation and less abundant changes of *Acetobacter* from both organically and conventionally fermented wine (Table 3 and Table S5). The increased abundance of *G. oxydans* during the organic fermentation process might explain the increased susceptibility to wine spoilage in wines that are produced using organic fermentation techniques. Overall, these results demonstrate that 16S rRNA gene sequencing technique can be used efficiently to obtain a detailed description of the bacterial population associated with grape juice and must and to discover novel microorganisms that might lead to wine spoilage. This ability will allow wine makers to prevent losing revenues and investing in NGS technologies pose a promising avenue for wine makers, in particular as NGS has become a commodity and software for NGS data analysis is freely available. By comparing community dynamics of organically and conventionally fermented grape musts, we also observed that the population of *Pedobacter, Sphingomonas, Janthinobacterium, and Pseudomonas* were significantly higher in musts subjected to conventional than organic fermentation practices. It also appears that the bacterial population associated with the conventionally produced wine, experiences more significant community changes during the vinification process. This finding can be explained by the fact that commonly additives such as DAP have a significant effect on the indigenous bacterial population (Figure 1) and affect the community profile almost instantly. On the other hand, the increased community complexity of conventionally fermented must is less expected although it can also be explained by the affect of the additives that are employed in the conventional fermentation process. These additives appear to affect primarily phylogenetic groups that are undesired during the fermentation process and that dominate the prokaryotic community prior to their addition. Additionally, decreased community complexity and diversity in the organically fermented grape juice might be caused by the presence of indigenous yeasts on the skin of grapes that are not subjected to fungicide (i.e., SO_2_) treatments during the organic PDC fermentation. This antimicrobial affect by indigenous yeasts in bacteria during the fermentation process was reported previously (Lonvaudfunel et al., 1988; Henick-Kling and Park, 1994) and it is possible that a defined mixture of naturally occurring yeast strains might represent a highly sustainable approach for controlling the composition and temporal succession of the bacterial population during the fermentation process. In order to make such yeast mixtures effective they would need to include additional strains that are efficient against the wine spoilage bacteria (e.g., *Gluconobacter oxydans*) that appear to be little affected by currently known indigenous grape skin yeasts.

Previously it was reported that winery surfaces were dominated by non-fermentation-related bacteria (i.e., *Pseudomonas, Comamonadaceae, Flavobaterium, Enterbacteraceae, Brevundimonas*, and *Bacillus*). Accordingly, we detected *Pseudomonas, Comamonadaceae, Enterbacteraceae*, and *Bacillus* during both organic and conventional fermentation (Table S6). The population of *Pseudomonas* and *Comamonadaceae* are larger at the early stage of conventional fermentation (2 days), which suggests that *Pseudomonas* and some members of *Comamonadaceae* originated from conventionally vinification process or their growth was not instantly inhibited by addition of SO_2_ prior to conventional vinification. The other possibility might be that the growth of *Pseudomonas* and some members of *Comamonadaceae* was suppressed by antimicrobial components produced by indigenous yeasts associated with organically fermented wine. *Enterbacteraceae*, a dominant family from grapevine (Pinto et al., 2014), is extremely abundant during PDC fermentation (about 85% in both samples), with a rapid population decrease during conventional fermentation (5% at day 2), this might be caused by addition of SO_2_. A less significant decrease was observed during organic fermentation [73% (day 2), 34% (day 10), 13% (day 13)], which might also be explained by the antimicrobial activity of an indigenous yeast that might have been associated with the grapes.

In this study, we obtained a more detailed understanding of the temporal succession of the bacterial population and associated changes of the wine chemistry during conventionally and organically fermented grapes using NGS technologies, which could not be studied with less sensitive molecular approaches (i.e., PCR-DGGE). The sequences generated during this study were deposited in NCBI's short read archive using the study accession number SRP058864. In summary, these results suggest that there are temporal changes in the bacterial population that is associated with the fermentation process and that these populations might contain microorganisms that have until today not been linked with the fermentation process. Further comprehensive study of how the bacterial species of wine interact and how the microbial community dynamics correlated with grape must and wine components during the fermentation process will be of great value for developing improved methods to control wine quality.

## Author contributions

Conceived and designed the experiments: MH. Performed the experiments: MH, HP, EH, SK, and SS. Generated and analyzed the data: MH, HP, EH, SK, RD, and SS. Wrote the paper: MH, HP, EH, and TH.

### Conflict of interest statement

The authors declare that the research was conducted in the absence of any commercial or financial relationships that could be construed as a potential conflict of interest.
