# Supplementary material for: Insights into the bacterial community and its temporal succession during the fermentation of wine grapes
Source: Front Microbiol. 2015 Aug 18;6:809. doi: 10.3389/fmicb.2015.00809 (PMC4539513; doi:10.3389/fmicb.2015.00809)
Supplement: Supplementary file 1 [file Table1.DOCX]

**Supplemental Table S1:** Barcode sequences.
