# Supplementary material for: Insights into the bacterial community and its temporal succession during the fermentation of wine grapes
Source: Front Microbiol. 2015 Aug 18;6:809. doi: 10.3389/fmicb.2015.00809 (PMC4539513; doi:10.3389/fmicb.2015.00809)
Supplement: Supplementary file 4 [file Table4.DOCX]

**Table S4.** Community composition of organic and conventional wine microbiome at the species level.
