# Supplementary material for: Insights into the bacterial community and its temporal succession during the fermentation of wine grapes
Source: Front Microbiol. 2015 Aug 18;6:809. doi: 10.3389/fmicb.2015.00809 (PMC4539513; doi:10.3389/fmicb.2015.00809)
Supplement: Supplementary file 6 [file Table6.DOCX]

**Table S6.** Relative abundance of *Enterobacteriaceae, Comamonadaceae, Pseudomonas* and *Bacillus*

*(f) indicates family

**(g) indicates genus
