# Supplementary figures and images for: Insights into the bacterial community and its temporal succession during the fermentation of wine grapes
Source: Front Microbiol. 2015 Aug 18;6:809. doi: 10.3389/fmicb.2015.00809 (PMC4539513; doi:10.3389/fmicb.2015.00809)

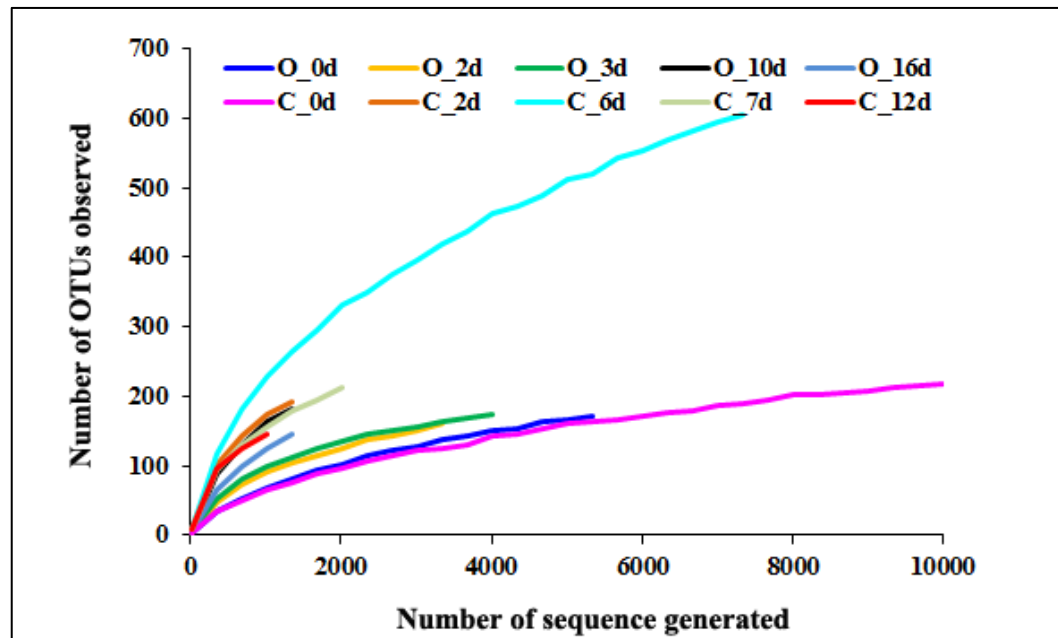

Supplemental Figure S1. Rarefaction curves

Supplement: Supplementary file 7 [file Image1.PDF]
